# Protein Array-Based Approach to Evaluate Biomarkers of Beef Tenderness and Marbling in Cows: Understanding of the Underlying Mechanisms and Prediction

**DOI:** 10.3390/foods9091180

**Published:** 2020-08-26

**Authors:** Mohammed Gagaoua, Muriel Bonnet, Brigitte Picard

**Affiliations:** National Research Institute for Agriculture, Food and Environment, Université Clermont Auvergne, VetAgro Sup, UMR Herbivores, F-63122 Saint Genès Champanelle, France; muriel.bonnet@inrae.fr

**Keywords:** meat tenderness, fat, proteomics, proteins, enzymes, quantification, biological pathways, chemometrics

## Abstract

This study evaluated the potential of a panel of 20 protein biomarkers, quantified by Reverse Phase Protein Array (RPPA), to explain and predict two important meat quality traits, these being beef tenderness assessed by Warner–Bratzler shear force (WBSF) and the intramuscular fat (IMF) content (also termed marbling), in a large database of 188 Protected Designation of Origin (PDO) Maine-Anjou cows. Thus, the main objective was to move forward in the progression of biomarker-discovery for beef qualities by evaluating, at the same time for the two quality traits, a list of candidate proteins so far identified by proteomics and belonging to five interconnected biological pathways: (i) energy metabolic enzymes, (ii) heat shock proteins (HSPs), (iii) oxidative stress, (iv) structural proteins and (v) cell death and protein binding. Therefore, three statistical approaches were applied, these being Pearson correlations, unsupervised learning for the clustering of WBSF and IMF into quality classes, and Partial Least Squares regressions (PLS-R) to relate the phenotypes with the 20 biomarkers. Irrespective of the statistical method and quality trait, seven biomarkers were related with both WBSF and IMF, including three small HSPs (CRYAB, HSP20 and HSP27), two metabolic enzymes from the oxidative pathway (MDH1: Malate dehydrogenase and ALDH1A1: Retinal dehydrogenase 1), the structural protein MYH1 (Myosin heavy chain-IIx) and the multifunctional protein FHL1 (four and a half LIM domains 1). Further, three more proteins were retained for tenderness whatever the statistical method, among which two were structural proteins (MYL1: Myosin light chain 1/3 and TNNT1: Troponin T, slow skeletal muscle) and one was glycolytic enzyme (ENO3: β-enolase 3). For IMF, two proteins were, in this trial, specific for marbling whatever the statistical method: TRIM72 (Tripartite motif protein 72, negative) and PRDX6 (Peroxiredoxin 6, positive). From the 20 proteins, this trial allowed us to qualify 10 and 9 proteins respectively as strongly related with beef tenderness and marbling in PDO Maine-Anjou cows.

## 1. Introduction

During the last two decades, OMICs techniques, especially proteomics, have been applied by meat scientists to understand the modifications occurring in post-mortem muscle in an attempt to explain the variation in several meat quality traits [1,2,3]. Further, proteomics allowed the identification of putative biomarkers (for review: [4,5,6]), with the objective of predicting the potential quality and also of proposing a molecular test for the beef industry [7]. Overall, two-dimensional gel electrophoresis combined with mass spectrometry was efficiently used to map and characterize bovine muscle proteins [8]. The main protein biomarkers so far identified belong to myriad interconnected pathways, such as structure and contraction, heat stock proteins, energy metabolism including the glycolytic and oxidative pathways, oxidative stress, transport, binding and signaling, apoptosis, and proteolysis, including endogenous muscle inhibitors such as serpins, cystatins and calpastatins [4,5,9,10].

Among the most investigated beef qualities using proteomics, tenderness has gained the most attention [11,12]. Beef tenderness is considered worldwide to be one of the most critical quality attributes for consumers and for re-purchase decisions. However, with beef tenderness being a multifactorial trait, it is highly variable, and is impacted by both intrinsic and extrinsic factors measurable along the continuum from farm-to-fork [13,14]. At the carcass and muscle levels, the intramuscular fat (IMF) content, also termed marbling, impacts tenderness variation and is considered as an important driver of beef palatability [15,16]. Proteomics was also applied to identify potential biomarkers of IMF and to address differences in adiposity [17] under several factors, such as breed [18], rearing practices [19], individual variability [20,21,22] and distinct stages of IMF development [23,24].

Based on the work by Rifai et al. [25], we recently detailed the process of meat quality biomarker discovery that should be followed so as to identify, evaluate and validate protein biomarkers at the research/industry scale [4,26]. This process is composed of six main steps that are discovery/identification, qualification, verification, research assay optimization, industrial validation and commercialization. In fact, the products of the discovery phase are lists of 10 to 100 proteins with different abundances between two compared situations or conditions (i.e., tender versus tough, or lean versus fat, etc.). Thanks to the current developments in the high-throughput techniques, several research groups have been able to move forward in the process of beef tenderness and IMF (marbling) biomarkers discovery by assaying fast techniques for the qualification of biomarkers in a few animals, which entails the confirmation of the differential abundances of the proteins using a method different from the one used for the discovery step. Thus, some shortlisted biomarkers were tested using immune-based techniques such as western blotting [27,28,29], Dot-Blot [30,31,32,33,34] and Reverse Phase Protein Array (RPPA) [35,36], or using label-free gel mass spectrometry tools, namely Selected Reaction Monitoring (SRM) and the sequential window acquisition of all theoretical spectra (SWATH) [37]. More recently, a combination of RPPA and Parallel Reaction Monitoring (PRM) [26] was used to both qualify and verify the reliability of a set of 10 proteins for predicting tenderness and marbling. Therefore, this study was designed to check the ability of a previous list of 20 protein candidate markers quantified by the RPPA technique to discriminate both beef tenderness and IMF in a large database of PDO Maine-Anjou cows, comprising 188 animals. It further aimed to qualify the most robust candidates that are predictors of both tenderness and marbling whatever the statistical method. The data of this trial will further increase our understanding of the role played by the qualified proteins in these two important beef qualities, to propose in the future generic biomarker-based tools for the early sorting of carcasses to meet consumer and industrial expectations.

## 2. Materials and Methods 

### 2.1. Experimental Designs, Cows Handling and Slaughtering

This trial was conducted using two replicate groups of 110 and 78 animals for a total of 188 Protected Designation of Origin (PDO) Maine-Anjou cows representative of the “Rouge des Prés” breed (for details on the experimental designs refer to Gagaoua, et al. [38] and Picard et al. [19]). The Rouge des Prés breed has, since 2004, been approved to be used in France for PDO meat production, hence it has a special economic importance for the valorization of local breeds as it is a dual-purpose cattle used for both beef and milk. PDO is the name of a geographical region or specific area that is recognized by official rules to produce certain foods with special characteristics related to location. The PDO regulation covers agricultural products and foodstuffs that are produced, processed and prepared in a given geographical area using recognized know-how in this specific zone. The certification label PDO and other labels are required by the European Commission for assuring the authenticity of food products. 

The main characteristics of the PDO Maine-Anjou breed are an age at slaughter lower than 10 years, having calved at least once and a minimal carcass weight of 380 kg. In this trial, all the cows had these qualities, with average age at slaughter and carcass weight of 67.4 ± 13.9 months and 445 ± 676 kg, respectively. The cows originated from the north-western part of France and were collected from the same cooperative of livestock farmers located in the department of Maine-et-Loire. All the animals were slaughtered in industrial slaughterhouses (n= 110 at Elivia, Lion d’Angers, France and n = 78 at Charal, Sablé sur Sarthes, France) following the same protocol. The cows had free access to water before their slaughter but food was deprived for 24 h. The exsanguination from the jugular vein was performed after electrical stunning using a captive-bolt pistol. Slaughtering was performed in compliance with French welfare and by respecting EU regulations (Council Regulation (EC) No. 1099/2009). The carcasses were dressed according to standard commercial practices and between 30–50 min post exsanguination the carcasses were split in half then chilled for 24 h at 2–4 °C. None of the carcasses were electrically stimulated. Ultimate pH was recorded at 24 h post-mortem for all the carcasses using a pH meter equipped with a glass electrode, and none of them had pHu > 6.0 (the benchmark used to sort DFD carcasses). 

### 2.2. Muscle and Meat Steaks Sampling

*Longissimus thoracis* (LT) muscle samples, known as a mixed fast oxido-glycolytic muscle, were taken from the 5th rib of the left-hand side of each carcass 24 h post-mortem. This sampling time is in line with the outcome of the discovery and validation of protein biomarkers of bee quality traits aiming at a management/prediction of the potential quality of the carcasses early post-mortem. Therefore, in this study and for three reasons we used muscle samples taken at 24 h post-mortem. First, this is in line with previous proteomics investigations from our team for the PDO Maine-Anjou breed and other laboratories for other animal types. Second, the sampling at industrial level was only possible at this time due to the company facilities and technical considerations. Third, at this time, we expect that the dynamic properties of the muscle expressed by changes in few but important proteins belonging to the energy metabolic or heat shock proteins pathways are less variable. Thus, the first part, free of connective tissue, was subsequently frozen in liquid nitrogen and stored at −80 °C until protein extractions for the quantification of the protein biomarkers using the RPPA technique. The second part of the sample was cut into 1–2 cm cross-section pieces, vacuum packed, and stored at −20 °C for intramuscular fat (IMF) content determination. The third part was cut into 20 mm thick steaks and placed in sealed plastic bags under vacuum and aged for 14 days at 4 °C (the usual and standard condition of ageing). Each loin sample was then frozen and stored at −20 °C awaiting Warner–Bratzler shear force (WBSF) measurements. 

### 2.3. Intramuscular Fat Content Determination

The amount of IMF on each muscle sample was determined using a Dionex ASE 200 Accelerated Solvent Extractor (Dionex Corporation, Sunnyvale, CA, USA) as previously described [38]. Briefly, 1 ± 0.001 g of meat powder was placed in a 22 mL extraction cell initially prepared with a cellulose filter and silicon balls. Then, petroleum ether at a temperature and pressure of 125 °C and 103 bars respectively was used for the extraction. The slurry containing both fat and petroleum ether was collected and transferred into a previously weighed evaporation vial (±0.001 g). After 15 min of evaporation, the vial was placed in a drying oven at 105 °C for 17 h and then weighed (±0.001 g) to determine the amount of IMF in the sample. The results were expressed as the % of IMF in fresh meat. 

### 2.4. Meat Tenderness Measurement by Warner–Bratzler Shear Force (WBSF)

For objective beef tenderness determination, Warner–Bratzler shear force (WBSF), known as a negative proxy of sensory tenderness, was measured according to Lepetit and Culioli [39] using an INSTRON 5944 testing machine. Briefly, the frozen steaks firstly thawed for 48 h at 4 °C, then were placed for 4 h in a thermostated bath at 18 °C before cooking on a double grooved plate griddle (SOFRACA, Morangis, France) set at 300 °C until the end-point temperature of 55 °C, which is the usual cooking temperature in France [40]. A temperature probe (Type K-Thermocouple, HI 98704, HANNA Instruments, Newark, NJ, USA) at the geometric center of the steak was used to control the end-point cooking temperature. After cooking, each steak sample was used to prepare five cores (1 cm × 1 cm × 4 cm) parallel to the longitudinal orientation of the muscle fiber. WBSF was assessed 2 or 3 times per core in order to obtain around 10 repetitions per sample. A 1 kN load cell and a 60 mm/min crosshead speed were used (universal testing machine, MTS, Synergie 200H). The force at the rupture during shear compression testing was expressed in N/cm^2^. 

### 2.5. Protein Extraction and Quantification

The muscle proteins were extracted by homogenization of the samples in “Precellys 24” tissue homogenizer (Bertin technologies, Saint Quentin-en-Yvelines, France) following the previously described protocol [41]. Briefly, 80 mg of frozen muscle were ground using 1.4 mm ceramic beads in an extraction buffer containing 50 mM Tris (pH 6.8), 5% glycerol, 2% SDS, 2 mM DTT, 2.5 mM EDTA, 2.5 mM EGTA, 1 × HALT Phosphatase inhibitor, Protease inhibitor cocktail complete MINI EDTA-free, 2 mM Na_3_VO_4_ and 10 mM NaF. The extracts were then boiled for 10 min at 100 °C, sonicated to reduce viscosity and centrifuged for 10 min at 25,000× *g*. The supernatants were collected and stored at −80 °C until use for protein assay and biomarkers quantification by RPPA.

Protein concentrations were determined with a commercial protein assay (Pierce BCA reducing agent compatible kit, ref. 23252, Thermo Scientific, Waltham, MA, USA) with bovine serum albumin (BSA) as standard. 

### 2.6. Reverse Phase Protein Array (RPPA) for Protein Biomarkers Quantification

The relative abundances of 20 protein biomarkers of tenderness and/or IMF (Table 1) were quantified by the Reverse Phase Protein Array [42] following exactly the same protocol recently detailed by our group on bovine muscle [26,35,36,41]. The 20 proteins belong to five biological pathways that are:

**(*i*) *Energy metabolic enzymes* (*n = 7*):** Malate dehydrogenase (MDH1), β-enolase 3 (ENO3), Retinal dehydrogenase 1 (ALDH1A1), Triosephosphate isomerase (TPI1), Phosphoglycerate kinase 1 (PGK1), Fructose-bisphosphate aldolase (ALDOA) and Glycogen phosphorylase (PYGB);

**(*ii*) *Heat shock proteins* (*n = 5*):** αB-crystallin (CRYAB), Hsp20 (HSPB6), Hsp27 (HSPB1), Hsp40 (DNAJA1) and Hsp70-1A (HSPA1A);

**(*iii*) *Oxidative stress proteins* (*n = 1*):** Peroxiredoxin6 (PRDX6);

**(*iv*) *Structural proteins* (*n = 5*):** MLC-1F (MYL1), Myosin heavy chain-IIx (MYH1), Troponin T, slow skeletal muscle (TNNT1), Titin (TTN) and Tubulin alpha-4A chain (TUBA4A);

**(*v*) *Cell death and protein binding* (*n = 2*):** Tripartite motif protein 72 (TRIM72) and Four and a half LIM domains 1 (FHL1).

After protein quantification by RPPA and for the determination of the relative abundance of each protein, the raw data were normalized using NormaCurve following the method described by Troncale et al. [43]. This is a SuperCurve-based method that simultaneously quantifies and normalizes RPPA data for fluorescent background per spot, the total protein stain and the potential spatial bias on the slide. Then, each RPPA slide was median-centered and scaled (divided by median absolute deviation). Further corrections to the sample loadings effects were performed individually for each array by correcting the dependency of the data for individual arrays with the median value of each sample over all 20 arrays using a linear regression. 

### 2.7. Statistical Analyses

The statistical analyses were carried out using XLSTAT 2018.3 (AddinSoft, Paris, France). Raw data were scrutinized for data entry errors and outliers using Smirnov–Grubb’s outlier test at a significance level of 5%. Then, all the data were normalized for replicates (experiment) and the factor related to the rearing practices of the animals [19,44]. This step was based on Z-scores, which represent the number of standard deviations for each observation relative to the mean of the corresponding replicate/condition. Therefore, after this transformation, the data had a mean of 0 and standard deviation of 1. Following this first step, three main statistical approaches were applied to the whole database to predict/explain each quality trait and evaluate the potential of each biomarker to contribute to its associated variation. 

***Correlations*:** Pearson correlation coefficients, based on the Z-scores, at the level of 5% were computed between WBSF values and IMF content with the 20 protein biomarkers.

***Clustering into WBSF and IMF classes*:** three unsupervised learning methods, which were (i) hierarchical cluster analysis (HCA), (ii) *k*-means and (iii) partitioning around medoids (PAM), were tested as previously described [45] to create meat quality classes of WBSF and IMF. For both quality traits, *k*-means gave the best results based on the average silhouette width (*Si*) criterion (Euclidean distance), allowing in the two cases three clusters or classes (*k* = 3) that we named tender, medium and tough for WBSF, and for IMF (marbling) fat, medium and lean. 

The value of *Si* ranges from −1 to +1, with observations that have a positive large *Si* being very well clustered. Those close to 0 (low *Si*) means that the observation lies between two clusters and those with negative *Si* are partitioned in the wrong cluster [46]. Afterward, variance analyses (ANOVA) were used to compare the protein abundances among the classes for each beef quality trait. Significant differences were performed using Tukey’s test at a significance level of 5% and were presented using heatmaps. Subsequently, principal component analyses (PCA) for each beef quality trait were carried out using the significant differential proteins (*p* < 0.05) to illustrate in a more complete picture the separation of the classes, and thus of the individuals and the distribution of the variables.

***Partial Least Squares regressions* (*PLS-R*):** to deepen our understanding of the mechanisms and identify the most robust explanatory protein biomarkers, Partial Least Squares regressions (PLS-R) were performed per beef quality trait to generate explanatory models using the optimal number of components in each case [47]. This is an appropriate tool to include all the 20 biomarkers in one model and identify those that had a biological and relevant significance using the criterion of the variable’s importance in the projection (VIP). This filter method based on VIP scores estimates the importance of each protein in the projection used in a PLS model. A protein with a VIP > 1.0 is considered important, thus highly influential in a given model, 0.8 < VIP < 1.0 is considered moderately influential, and any protein with VIP < 0.8 is less influential thus considered weak and rejected. For the selection of the variables, the jack-knife method was included in the PLS-R as a selective parameter. In this study, all proteins for which the VIP scores were above a threshold of 1.0 and 0.8 (highly and moderately influential proteins) were considered and then compared with those selected from correlation and variance analyses to be used in the future for validation on meat from PDO Maine-Anjou cows. 

## 3. Results

### 3.1. Pearson Correlation Analyses between the Biomarkers and Meat Quality Traits

The correlation coefficients computed between WBSF values and IMF content with the 20 protein biomarkers and for all the 188 cows are given in Table 2. 

For WBSF, 11 proteins were significantly (*p* < 0.05) correlated with this texture trait. Four proteins were from the energy metabolism pathway: MDH1, ENO3, PGK1 (all negative) and ALDH1A1 (positive). These were followed by three small heat shock proteins (sHSP): CRYAB, HSP20 and HSP27 (all positive). Three were structural proteins: MYL1 and MYH1 (both negative) and TNNT1 (positive). One protein from the last pathway, which was FHL1, was positively correlated with WBSF.

For IMF content, 10 proteins were significantly (*p* < 0.05) correlated, of which 2 were energy metabolic enzymes that are MDH1 (negative) and ALDH1A1 (positive), 3 were sHSP (CRYAB, HSP20 and HSP27) that were all positive, and 2 were structural proteins (MYH1 (negative) and TNNT1 (positive)). The PRDX6 from the oxidative pathway was positive, and two proteins from the last pathway, TRIM72 and FHL1, were respectively negatively and positively correlated with IMF content (Table 2).

From the above, eight proteins, MDH1, ALDH1A1, CRYAB, HSP20, HSP27, MYH1, TNNT1 and FHL1, were common to the two traits. Interestingly, sHSPs seemed from this analysis to be important biomarkers for both WBSF and IMF. 

### 3.2. Discriminant Biomarkers of WBSF and Marbling

In the attempt to evaluate discriminant biomarkers for both WBSF and IMF classes, the *k*-means algorithm, as the best clustering method, allowed the identification of three classes for each beef quality trait.

**Figure 1:**
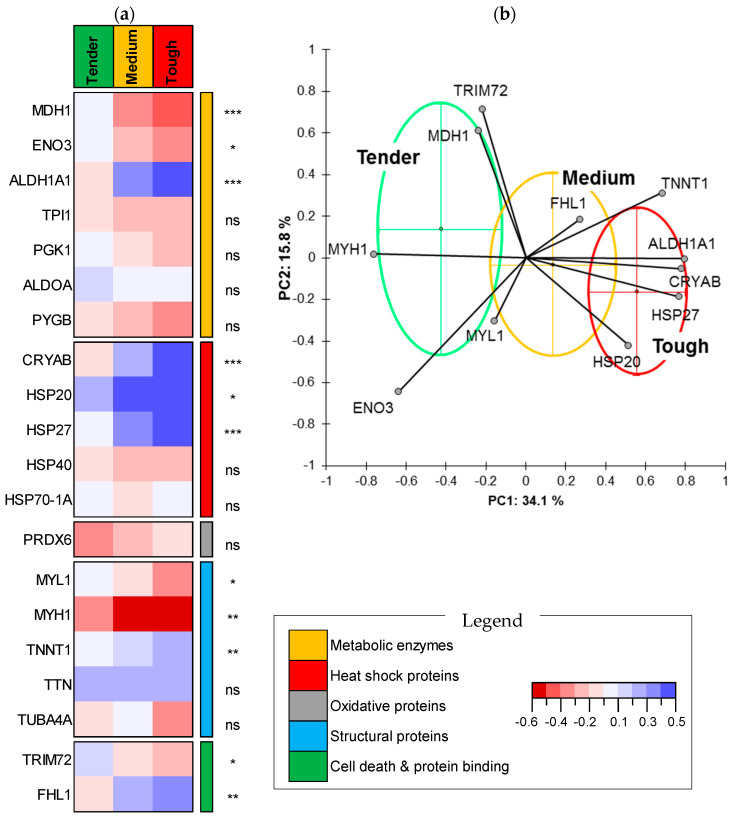
Protein biomarkers differing among the tenderness classes (Tender (*n* = 93), Medium (*n* = 71) and Tough (*n* = 24)). (**a**) Heatmap comparing the protein abundances among the three WBSF (tenderness) classes. Significance—ns: not significant; *: *p* < 0.05; **: *p* < 0.01; ***: *p* < 0.001. The proteins are given by their biological family following the legend. (**b**) Principal component analysis highlighting the distribution of the individuals of each tenderness class based on the 11 discriminant protein biomarkers. Individuals belonging to the same class are encircled in clusters using the corresponding schematic colors. The descriptive statistics of the three tenderness classes are as follows—**Tender class:** mean value of 32.96 ± 3.99 N/cm^2^ (CV, 12%), Min = 23.05 and Max = 38.76 N/cm^2^. **Medium class:** mean value of 44.74 ± 3.69 N/cm^2^ (CV, 8%), Min = 39.00 and Max = 52.22 N/cm^2^. **Tough class:** mean value of 61.18 ± 7.87 N/cm^2^ (CV, 13%), Min = 53.03 and Max = 81.49 N/cm^2^.

For WBSF, the *k*-means clustering of the 188 steaks of the PDO Maine-Anjou cows categorized them into tender (*n* = 93), medium (*n* = 71) and tough (*n* = 24) samples (Figure 1). The tender class has a mean value of 32.96 ± 3.99 N/cm^2^, a coefficient of variation (CV) of 12% and WBSF values ranging between 23.05 and 38.76 N/cm^2^. The medium tenderness class has a mean value of 44.74 ± 3.69 N/cm^2^, a CV of 8% and WBSF values ranging between 39.00 and 52.22 N/cm^2^. The tough class has a mean value of 61.18 ± 7.87 N/cm^2^, a CV of 13% and WBSF values ranging between 53.03 and 81.49 N/cm^2^. Comparison of the protein abundances of the 20 biomarkers, based on variance analysis among the three clusters, highlighted that 11 proteins were significantly different at the level of 5% (Figure 1a). Among them, three proteins were from the energy metabolism pathway, MDH1 and ENO3 being highly abundant in the tender class compared to the tough samples, and the inverse was found for ALDH1A1 (*p* < 0.001). The three sHSPs (CRYAB, HSP20 and HSP27) were all highly abundant in the tough compared to the tender class. Among the structural proteins, three proteins were discriminant, which were MYL1 and MYH1 (highly abundant in tender meat), and TNNT1 was found to be more abundant in the tough class. Finally, TRIM72 and FHL1 from the last pathway were respectively highly and less abundant in the tender class, compared to the tough class (Figure 1a). The main significant discriminant proteins were MDH1, ALDH1A1, CRYAB and HSP27 (*p* < 0.001), followed by MYH1, TNNT1 and FHL1 (*p* < 0.01), and then ENO3, HSP20, MYL1 and TRIM72 (*p* < 0.05). The projection of these WBSF discriminant proteins on a PCA allowed acceptable separation of the three WBSF classes (Figure 1b). The first two principal components (PC) explained around 50% of the WBSF variability, with most variation being explained by the first PC (34.1%). The cows characterized by tender meat, thus being in the tender class, were all loaded on the left, the medium were in the center, and the tough were on the right. A total of six proteins characterize the tough class and the remaining five proteins were higher in the tender class (Figure 1b).

**Figure 2:**
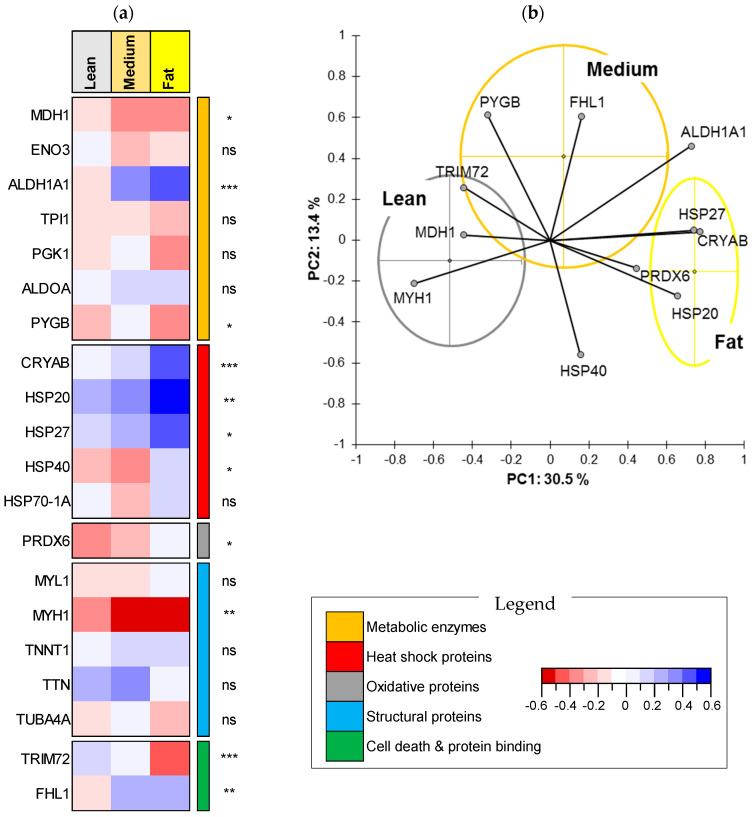
Protein biomarkers differing among the marbling classes (Fat (*n* = 28), Medium (*n* = 69) and Lean (*n* = 87)). (**a**) Heatmap comparing the protein abundances among the three IMF (marbling) classes. Significance—ns: not significant; *: *p* < 0.05; **: *p* < 0.01; ***: *p* < 0.001. The proteins are given by their biological family following the legend. (**b**) Principal component analysis highlighting the distribution of the individuals of each marbling class based on the 11 discriminant protein biomarkers. Individuals belonging to the same class are encircled in clusters using the corresponding schematic colors. The descriptive statistics of the three marbling classes are as follows—**Fat class:** mean value of 7.72 ± 1.58% (CV, 20%), Min = 6.34 and Max = 13.82%. **Medium class:** 4.72 ± 0.63% (CV, 13%), Min = 3.76 and Max = 6.11%. **Lean class:** 2.72 ± 0.62% (CV, 23%), Min = 0.45 and Max = 3.69%.

For IMF, the 188 steaks of the PDO Maine-Anjou cows were categorized into three marbling classes, as they were for WBSF, (Figure 2) namely fat (*n* = 28), medium (*n* = 69) and lean (*n* = 87). The fat class has a mean value of 7.72 ± 1.58%, a CV of 20% and values ranging between 6.34 and 13.82%. The medium fat class has a mean value of 4.72 ± 0.63%, a CV of 13% and IMF values ranging between 3.76 and 6.11%. The lean class has a mean value of 2.72 ± 0.62%, a CV of 23% and IMF values ranging between 0.45 and 3.69%. Eleven proteins were significantly different (*p* < 0.05) among the three marbling classes (Figure 2a). The main discriminant biomarkers were from the HSP superfamily, with a total of four proteins (CRYAB, HSP20, HSP27 and HSP40) that were all highly abundant in the fat class compared to the others (Figure 2a). This is followed by the energy metabolism pathway, with three proteins: MDH1 (high in lean class), ALDH1A1 (high in fat class) and PYGB (high in medium class). PRDX6 from the oxidative stress pathway and MYH1 from the structural pathway were respectively high and low in the fat class (Figure 2a,b). From the last family group, the two proteins TRIM72 and FHL1 were both different in their abundance among the marbling classes, being respectively low and high in the fat class. FHL1 was in this trial more abundant in the intermediate fat group. The main most significant discriminant proteins were ALDH1A1, CRYAB and TRIM72 (*p* < 0.001), followed by HSP20, MYH1 and FHL1 (*p* < 0.01), and then MDH1, PYGB, HSP27, HSP40 and PRDX6 (*p* < 0.05). The projection of these 11 discriminant proteins on a PCA allowed for separating efficiently the three marbling classes, especially the lean and fat samples (Figure 2b). The first two PC explained around 44% of the variation, with 30.5% in the first PC.

From the above, the clustering analysis allowed us to observe that eight proteins (MDH1, ALDH1A1, CRYAB, HSP20, HSP27, MYH1, TRIM72 and FHL1) are common for the two traits, and clearly delineate the beef quality classes. As for the correlation analyses, sHSPs seemed also to be important biomarkers for both WBSF and IMF.

### 3.3. Partial Least Squares for the Prediction of WBSF and Marbling Using the Panel of 20 Protein Biomarkers

**Figure 3:**
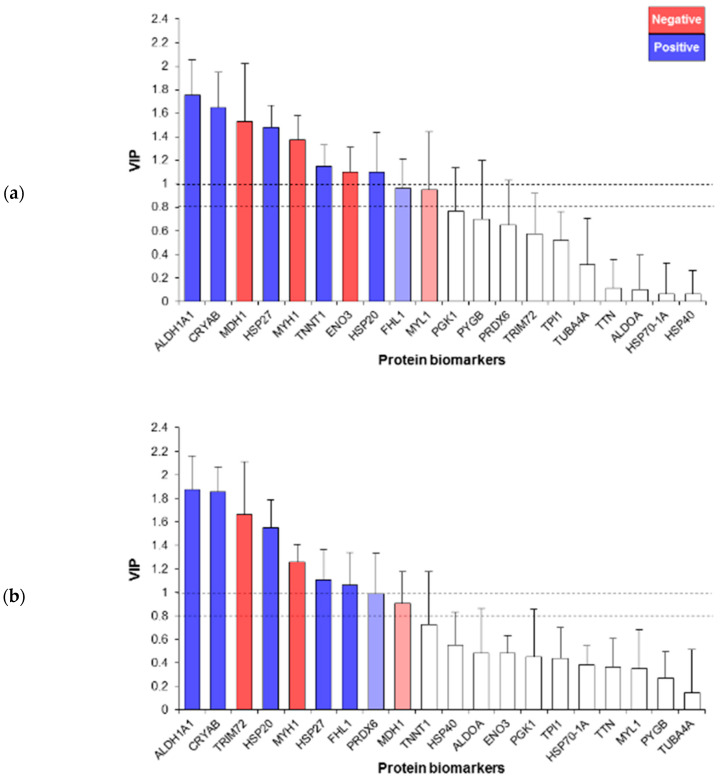
Partial least squares highlighting the protein biomarkers retained to explain (**a**) tenderness evaluated by WBSF and (**b**) IMF content (marbling) based on their variable importance in the projection (VIP). The proteins retained in positive and negative directions are shown in blue and red colors, respectively. For (**a**) WBSF, a total of 10 proteins were retained from which 8 had VIP > 1.0, and for (**b**) IMF, 9 proteins were retained, from which 7 had VIP > 1.0. A total of 7 proteins were common (MDH1, ALDH1A1, CRYAB, HSP20, HSP27, MYH1 and FHL1) in the two models to explain both WBSF and IMF variation.

The investigation using PLS-R of the relationships between the 20 protein biomarkers and the two beef quality traits evaluated on the 188 PDO Maine-Anjou cows generated explanatory models with the main drivers of their variation (Figure 3a,b). The WBSF model retained 10 proteins (Figure 3a). Among them, eight proteins had VIP values > 1.0 (ALDH1A1, CRYAB, MDH1, HSP27, MYH1, TNNT1, ENO3 and HSP20) and 2 hade 0.8 < VIP < 1.0 (FHL1 and MYL1). Three proteins for the energy metabolism, small HSP and structural proteins pathways, respectively, were retained. From the cell death and binding protein family, one protein, FHL1, was retained for WBSF. 

For IMF, the PLS-R generated an explanatory model with nine proteins (Figure 3a). Among the retained proteins, seven had a VIP value > 1.0 (ALDH1A1, CRYAB, TRIM72, HSP20, MYH1, HSP27 and FHL1) and two had 0.8 < VIP < 1.0 (PRDX6 and MDH1). 

For both PLS-R explanatory models, seven proteins were common (ALDH1A1, CRYAB, MDH1, HSP20, HSP27, MYH1 and FHL1) in explaining WBSF and IMF variations. Interestingly, for both quality traits models, the first ranked proteins with VIP values > 1.5 were ALDH1A1 and CRYAB (Figure 3a,b).

### 3.4. Summary of the Putative Common Protein Biomarkers from the Three Statistical Methods

**Figure 4:**
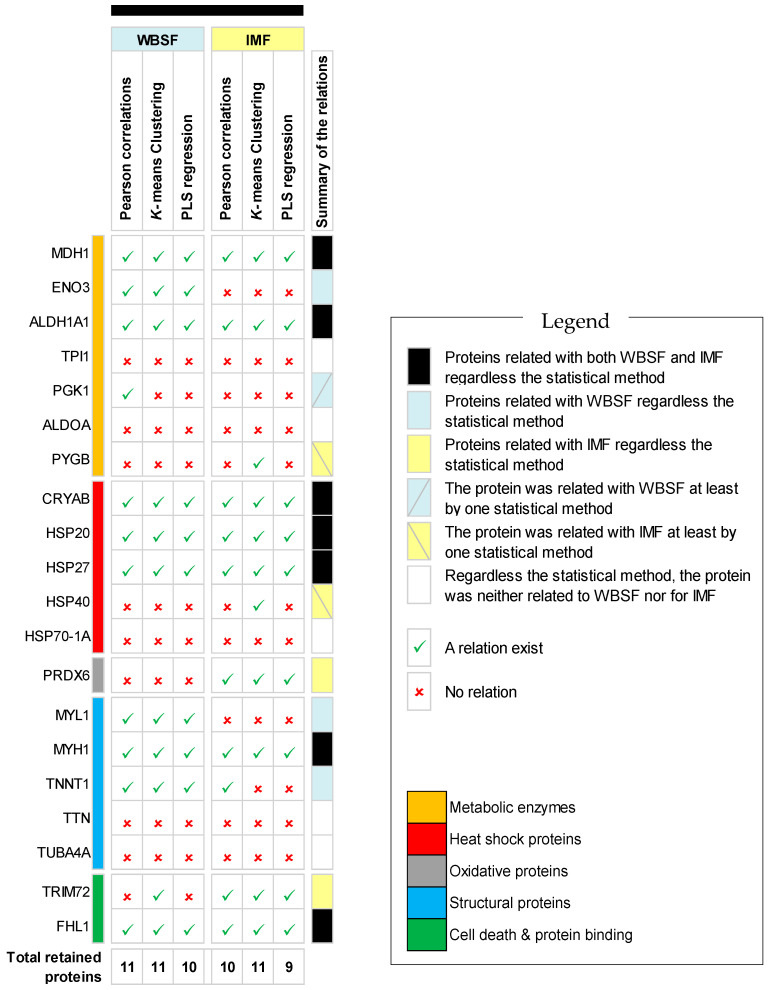
Summary of the evaluation of the 20 protein biomarkers quantified by RPPA using the three statistical methods (Pearson correlations, *k*-means clustering and Partial Least Squares regressions (PLS-R)) to explain/predict WBSF and IMF content on the *Longissimus thoracis* muscle of the 188 PDO Maine-Anjou cows.

The summary of the proteins retained from the 20 panel biomarkers, based on the results of the three statistical methods presented above to explain/predict WBSF and IMF content, is given in Figure 4. Overall, irrespective of the statistical method and quality trait and based on the variable importance of the proteins in the models, their accuracy in discriminating the quality groups and their significant associations with WBSF and IMF, seven biomarkers were common, including three small HSPs (CRYAB, HSP20 and HSP27), two energy metabolic enzymes from the oxidative pathway (MDH1 and ALDH1A1), the structural protein MYH1 and the multifunctional protein FHL1. Further, for WBSF three more proteins were retained whatever the statistical method, among which two were structural proteins (MYL1 and TNNT1) and one was a metabolic enzyme (ENO3). For IMF, two proteins were retained whatever the statistical method, these being TRIM72 and PRDX6.

## 4. Discussion

This trial aimed to evaluate the potential of 20 protein biomarkers, previously identified by proteomics to be potential markers of beef tenderness and marbling [4,11,12,17,19,22,26,34,35,48,49,50] and belonging to five interconnected biological pathways ((i) energy metabolic enzymes, (ii) heat shock proteins (HSPs), (iii) oxidative stress, (iv) structural proteins and (v) cell death and protein binding) to explain/predict two important beef quality traits for both the consumers and the meat sector (meat tenderness measured by WBSF and the marbling evaluated by the percentage of IMF content). The list of proteins was selected for qualification in this trial based on two main criteria: (i) association of the protein with the quality traits from previous studies, and (ii) validation of an antibody against the protein for its quantification by RPPA. Therefore, we used the immunological RPPA technique for their semi-quantification, and applied three statistical methods to explain the variability of each quality trait, being Pearson correlations to assess the type of associations with the quality traits, unsupervised learning to perform a clustering of the quality traits and determine the main protein splitters, and Partial Least Squares regressions (PLS-R) to propose the first overall regressions models and identify the main predictor proteins based on their importance in the model. This firstly correlates the relative abundances of the candidate protein markers with WBSF and IMF, and secondly describes the consistencies and differences for the two traits. 

This study is the first to use such a large database of 188 cows to perform this qualification/evaluation step on the selected 20 protein biomarkers, hence allowing us to move forward in the progression of biomarkers discovery for beef qualities [4] by evaluating at the same time two beef quality traits. From the evaluated list of proteins, we revealed (regardless of statistical method) that certain biomarkers are robust for both beef qualities of PDO Maine-Anjou cows. A robust biomarker is the protein that is identified in this study to be related with both beef quality traits whatever the statistical method. In the following sections, the proteins common to the two traits are discussed together, and those that were trait-dependent are presented separately. The biological pathways behind the associations identified between the two phenotypes and the proteins were further briefly presented. The relationships between the robust evaluated protein biomarkers and the two beef quality traits showed that the most tender meat of PDO Maine-Anjou cows had higher abundances of glycolytic enzymes, such as ENO3, and of fast contractile proteins such as MYL1 and MYH1, while they had lower abundances of slow contractile proteins such as TNNT1, and lower abundances of small HSPs with higher abundances of FHL1. These relationships are consistent with each other and are in accordance with the results of Couvreur et al. [51] based on the contractile and metabolic properties of the *Longissimus thoracis* muscle. Furthermore, the contractile properties of the *Longissimus thoracis* muscle from PDO Maine-Anjou cows have been associated with a specific fiber type composition, as the muscle contains a very low proportion of IIX fibers and a higher proportion of IIA fibers compared to the French beef breeds [38,51,52,53]. Consequently, its glycolytic metabolism is very low [51,52]. This demonstrates that for a slow oxidative type of muscle, the most tender are the less slow oxidative and the most fast glycolytic, as observed for Aberdeen Angus [54] or Chianina breeds [49].

### 4.1. Common Biomarkers Explaining the Variation in WBSF (Tenderness) and IMF (Marbling)

The data of the present trial showed that the common proteins between tenderness and IMF are usually inversely associated, except for FHL1 which was positive. WBSF was weakly and negatively correlated with IMF (r = +0.16; *p* = 0.032). The fatter meat samples are those containing higher proportions of proteins of the slow oxidative type, such as TNNT1 and small HSPs, and low proportions of the fast glycolytic type, such as MYH1, which is consistent with previous results about biomarkers of adiposity from the same breed [22,26]. From these data, it seems that the fatter meat samples do not lead to the tenderest beef. Thus, irrespective of the quality trait and the statistical method, seven proteins were robustly related with the WBSF and IMF content of PDO Maine-Anjou cows (Figure 4). 

Without any surprise and in line with the results presented above, the superfamily of heat shock proteins, specifically the small HSP members, seemed to be just as good and important biomarkers for both beef qualities. Indeed, CRYAB, HSP20 and HSP27 were positively related in this trial with WBSF (negatively with tenderness), and so with toughness and IMF content. An inverse relationship between the abundance of small HSPs and tenderness was reported in the *Longissimus thoracis* muscle of the Angus breed [54]. It is worthwhile to note that the *Longissimus thoracis* muscles of the PDO Maine-Anjou breed investigated in this study, as well as those of Aberdeen Angus, were described as having more oxidative metabolisms with high amounts of fat content [18,38,52,54]. The slow oxidative type fibers are known to contain high levels of sHSP proteins [52], as also evidenced by Golenhofen et al. [55] in rats, showing more HSPs in slow oxidative muscles. 

The involvement of these chaperones in post-mortem muscle, and thus in its conversion into meat, was reported by many earlier studies (for review: [5,11,12,56]). This is in agreement with the onset of apoptosis, the first phase in the conversion of muscle into meat, involving major biochemical and structural changes that influence not only the meat tenderization process but also the homeostasis of the post-mortem muscle [9,57]. Small HSP proteins were thought to stabilize and ensure the correct folding of newly synthesized proteins, or help refold proteins altered by cell stress to protect them against metabolic disorders and ischemia [58]. For example, small HSPs can bind to myofibrils [59,60,61], thereby protecting skeletal muscle through structural protein complexes, which partly explain the high abundance observed in the tough meat class. Accordingly and during the process of apoptosis, CRYAB is able to negatively regulate Cytochrome c and Caspase-8, hence inhibiting the executor caspase 3 [62,63]. For instance, any increase in the level of CRYAB (maybe also the other small HSPs) leads to less muscle structure degradation, which would produce tough meat [48]. In support of their important roles in meat tenderization, small HSPs were identified by several previous proteomics and under different conditions as good biomarkers for the two qualities investigated in this trial [6,17], or other sensory qualities of beef such as color [5] and pH decline [52,64,65]. CRYAB, which was ranked second in the PLS-R models, is a well-known beef tenderness biomarker [4,29,50,66,67,68,69], also recently identified [22] using gel-based and gel-free proteomics methods for IMF of the same breed using PRM and antibody-based proteomics [26]. HSP27 was reported by proteomics in several studies for beef tenderness [4,27,29,35,66,67,70], and as a consistent biomarker of adiposity (marbling) in the few proteomics that investigated this trait [18,20,22,24,71]. An absolute quantification of HSP27 in PDO Maine-Anjou cows confirmed a higher abundance in the less tender *Longissimus thoracis* muscle [26], as observed in this study. For HSP20, this study is the first to identify it as a biomarker for marbling, but it is already connected by several authors to beef tenderness [4,34,35,49,66,67,68]. Worthy of note is that in humans and among the HSP protein members, HSP20 was found by DeLany et al. [72] to be the most up-regulated chaperone during the differentiation of human adipose-derived stem cells into mature adipocytes. 

In line with the above, ALDH1A1, a metabolic enzyme that is ranked first in the PLS-R models regardless of the quality trait, and is also significantly correlated with both traits and discriminates groups of tenderness and IMF, appeared as the most robust biomarker in this study with CRYAB. ALDH1A1 has been already identified as a biomarker of adiposity of PDO Maine-Anjou [22,26]. The authors proposed that any increase in this protein would mediate an increase in CRYAB and maybe in other small HSPs thanks to the retinoic acid content [73]. Further, the same authors postulated a mechanism in PDO Maine-Anjou [22,26], suggesting that a higher abundance of CRYAB resulting from a high proportion of slow fibers may sustain the elevated oxidative metabolism of marbled muscle, which may partly explain the important role that ALDH1A1 and CRYAB play in the determination of both WBSF and IMF content. ALDH1A1 was identified as a good biomarker of beef tenderness in different breeds and muscles [35,68,74,75], and was also connected to several color parameters [5], especially redness (a*) [41] and metmyoglobin-reducing activity [76]. These last parameters are associated with the oxidative metabolism resulting from the fiber composition of the *Longissimus thoracis* muscle from Rouge des Près cows (with a very low proportion of fast glycolytic fibers [52]). It is worthy of mention that ALDH1A1 is able to protect cells against the cytotoxic effects of various aldehydes [77], which are generated in the cytosol by lipid peroxidation [78] and not cleared due to the cessation of blood flow. 

The second metabolic enzyme, the cytosolic MDH1, is a member of the malate dehydrogenase enzymes very important for both gluconeogenesis and the Krebs cycle, and therefore plays a crucial role in energy and cellular metabolism [79]. In adipocytes, including muscular adipocytes, MDH1 is involved in the reduced nicotinamide adenine dinucleotide phosphate (NADPH) supply for de novo fatty acid synthesis, and is considered as a lipogenic enzyme. It was retained in this study in agreement with earlier studies. For instance, it was reported (as a biomarker of beef tenderness in bulls) to be positive in two studies [28,54] and negative in another [4]. MDH1 was also shown as a good predictor of *Longissimus thoracis* tenderness variability (in a few animals) when quantified by PRM [26]. Moreover, malic enzyme activity (MDH1) and IMF quantitative values in the *Rectus abdominis* and *Semitendinosus* muscles from Limousin, Angus and Japanese Black cross Angus steers were shown to be positively correlated [80]. Similar positive correlations between MDH1 and IMF were also observed when the abundance of MDH1 was assayed by absolute PRM quantification in both *Longissimus thoracis* and *Semitendinosus* muscles, but not by RPPA [26]. In the present study, using a larger number of animals and using the RPPA technique, an expected link between MDH1 and IMF was revealed by three mathematical methods. However, an unexpected negative correlation between MDH1 abundance and IMF probably arose from the relative and normalized abundance of MDH1 in the present study, making difficult the comparison between the current and previous results. This warrants further investigation in order to understand and describe the link between the MDH1 protein’s abundance and IMF depending on the methodological specificities of both the protein quantification method and data processing.

MYH1 is the only structural protein that was related to both WBSF and IMF content whatever the statistical method (Figure 4). Changes in the cytoskeletal proteins have been shown and investigated for decades to play a role in meat tenderization [81], and are also evidenced by proteomics [4,6,11,82]. Further, myosin fibers play pivotal roles from fetal life to the slaughter of cattle (for review: [83]). MYH1 is the gene that encodes the fast glycolytic IIX fibers. In this study, the low abundance of MYH1 in the lean marbling class is in agreement with the earlier data reported for breeds of French origin, characterized by high proportions of fast glycolytic IIX fibers [84]. Cytoskeletal proteins were proposed to participate in IMF deposition [22,85,86], and MYH1 has already been identified by proteomics as a negative biomarker of adiposity [71]. MYH1 was revealed in several studies and under different factors as a good biomarker of beef tenderness [4,34,35,54,67,87,88]. As reported by Picard et al. [54] and Gagaoua et al. [34], the direction of its relationships with tenderness depends on muscle type, breed, end-point cooking temperature and origin of the panelists. In fact, in muscles with a low proportion of fast glycolytic fibers, such as the *Longissimus thoracis* muscle in breeds like Rouge des Prés or Aberdeen Angus, MYH1 was already reported to be positively related with tenderness. On the contrary, in French beef breeds in which the *Longissimus thoracis* muscle contains a high proportion of IIX fibers, the relationship with tenderness is negative. The identification of MYH1 as an important biomarker agrees with the theory stating that muscles with high proportions of fast fiber types are more susceptible to early post-mortem proteolytic degradation [89]. This can be further explained by their susceptibility to post-mortem glycolysis, hence leading to more tender than tough meat [83].

The seventh and last protein retained in this trial for the two beef quality traits was FHL1, known as a multifunctional protein regulating metabolism, cell proliferation, gene transcription and apoptosis [90]. This protein can further interact with metabolic enzymes as a response to the oxidative stress in muscle as well as hypoxia [90], thereby explaining its tendency to be projected with proteins characterizing slow oxidative properties, such as ALDH1A1 and TNNT1. To our knowledge, FHL1 was reported as a biomarker of beef tenderness in the *Longissimus thoracis* muscle in four previous studies [35,91,92,93], and of marbling in three studies [22,26,94]. We suggest that it is mainly via the regulation of calcium homeostasis [95] that FHL1 plays a role in beef tenderness determination. Indeed, Ca^2+^ ions contribute to the regulation of the energy metabolism pathways [96], as they affect the enzymatic speed of several crucial metabolic enzymes [11,12]. This is consistent with our results showing modifications of the abundances of the proteins ALDH1A1, MDH1 and ENO3. Calcium concentrations have also been implicated as initiators of apoptosis via some signaling pathways in skeletal muscle [12,97]. For example, apoptosis was documented to affect the integrity of the skeletal muscle, through the modification of Ca^2+^ flux during ageing and its consequences on protein proteolysis involving ultra-structural modifications. This can be supported in this trial by the robust association of TNNT1, MYL1 and MYH1, which are all proteins of structure. Further, FHL1 belongs to this last group of proteins and was described as an activator of the myostatin signaling pathway in skeletal muscle by promoting muscle atrophy [98]. This would explain the variation in the different muscles fibers and the involvement of MYH1 (further details, see [83]). For marbling, the mechanism would also be partly through the activation of myostatin signaling [98]. It is worthy of note that in beef, FHL1 was further related negatively with the lightness (L*) and positively with the redness (a*) color parameters [41].

### 4.2. Biomarkers Specific to WBSF

Irrespective of the statistical method, three proteins were found to be robust and specific to WBSF (Figure 4), these being two structural proteins (MYL1 and TNNT1) and one glycolytic enzyme (ENO3). The positive association of MYL1 with tenderness (negative with WBSF) is in agreement with several earlier studies on cows [4,38], steers [68,99] and young bulls [54,66,70]. MYL1 is considered as an indicator of proteolysis [81] and is prone to phosphorylation, a reaction playing a pivotal role in muscle to meat conversion [82,100]. It is worthy to consider that this phosphorylation is induced through sarcoplasmic reticulum Ca^2+^ release in a concentration-dependent manner. TNNT1 is a slow isoform of the troponins complex that is involved in the regulation of muscle contraction [81]. The release of TNNT members, including TNNT1, has been extensively studied and they have been considered as important substrates of the endogenous muscle proteolytic systems [11,12,82]. TNNT members were thought to be easily degraded by calpains during the aging period of muscle. In fact, several proteomics trials identified TNNT1 as a good biomarker of beef tenderness [4,66,99,101]. Its negative association with tenderness in this trial agrees with what we know from the literature [102], as recently evidenced by a meta-proteomics on different muscles and genders [4], highlighting inverse relationships. In the present study, the identification of TNNT1 is consistent with the positive relation observed between the fast glycolytic isoform of MYH1 and the glycolytic enzyme ENO3, and tenderness. 

ENO3 is the last protein we identified as robustly related to tenderness. Its identification as a positive biomarker of beef tenderness in this study on PDO Maine-Anjou cows and several previous proteomics on beef [4,35,67,87,92,103] points to its importance, as it is especially described as being a key moonlighting enzyme associated with hypoxic conditions and stress [104]. Enolase is a cytosolic enzyme responsible for the conversion of 2-phosphoglycerate into phosphoenolpyruvate, thereby playing an important role in pH decline and post-mortem metabolism [41]. Indeed, ENO3 induces glucose metabolism under hypoxic conditions [105], hence enacting a cellular stress response to the deprivation of oxygen supply and glucose levels. 

### 4.3. Biomarkers Specific to IMF

Two proteins, Peroxiredoxin 6 (PRDX6) and Tripartite motif protein 72 (TRIM72), appeared to be robustly related as biomarkers of marbling (Figure 4). PRDX6 was already identified as a good biomarker of beef tenderness [35,49,50,68,92], color [5] and pH decline [33]. PRDX6 is a bi-functional protein with both phospholipase A2 (PLA2) and glutathione peroxidase activities, which is expressed in nearly all tissues and protects cells against oxidative stress [106]. Earlier studies reported the high abundance of PRDX6 in Aberdeen Angus (young bulls and cross sired steers), known as a marbled breed compared to young Limousin bulls [32] and Belgian Blue sired steer [18]. PRDX6 might further play a role through its PLA_2_ activity via the catalysis of the hydrolysis of the acyl group at the sn-2 position of glycerophospholipids, with a specific link to phosphatidylcholine to release free fatty acids and a lysophospholipid [106], hence partly explaining its relation with IMF. Further, using PRDX6 knockout mice, Arriga et al. [107] demonstrated that PRDX6 modulates the link between glycemic and lipogenic metabolisms, thereby playing a pivotal role in fat deposition. 

TRIM72, also termed MG53 (Mitsugumin 53), is a signaling protein that acts as a sensor of oxidation on membrane damage [108], including playing a crucial role in the muscle membrane repair process. Its negative relationship with IMF would be partly explained by its implication in the clearance of harmful agents collected under the cell death process and lipid oxidation. TRIM72 was reported as a negative biomarker of beef tenderness [4,35,74], and thus one can suggest that a reduced cell death phase in tough meat occurred. TRIM72 was further identified as a biomarker of beef color Lightness (L*) [41]. The identification of TRIM72 as a biomarker of marbling in PDO Maine-Anjou would be supported by its involvement in the negative feedback regulation of myogenesis, by targeting the insulin receptor substrate-1 [109]. Further investigations are warranted in order to clarify the exact role of TRIM72 in the muscle to meat conversion, including the role it plays in marbling.

## 5. Conclusions

This study allowed us to qualify, on *Longissimus thoracis* muscle and using the immune-based RPPA technique and different statistical methods, the potential of a list of 20 protein biomarkers to explain the variation of two important beef quality traits of PDO Maine-Anjou cows. These were tenderness, measured by the instrumental method WBSF, and the marbling of the carcasses as evaluated by IMF content. This study is the first to propose 10 and 9 proteins as robust candidate biomarkers of WBSF and IMF, respectively, in Rouge des Prés cows regardless the statistical method. Seven proteins are of specific interest, as they are related to both traits. They are, in the order of importance, ALDH1A1, CRYAB, HSP27, HSP20, MYH1, FHL1 and MDH1. These proteins belong to the superfamily of heat shock proteins, energy metabolism (especially the oxidative pathway), and the structural proteins (including FHL1) playing roles in cell death, metabolism and the regulation of calcium homeostasis. Our findings further highlight that similarities exist in the biological pathways underpinning tenderness and marbling determination. 

## Figures and Tables

**Table 1:** List of the 20 protein biomarkers quantified using the Reverse Phase Protein Array (RPPA) technique ^1^.

Protein Biomarkers Name (*Gene*)	Uniprot ID	Monoclonal (Mo) or Polyclonal (Po) Antibodies References	Antibody Dilutions
**Energy metabolic enzymes**			
Malate dehydrogenase (*MDH1*)	Q3T145	Mo. anti-pig Rockland 100-601-145	1/1000
β-enolase 3 (*ENO3*)	Q3ZC09	Mo. anti-human Abnova Eno3 (M01), clone 5D1	1/30,000
Retinal dehydrogenase 1 (*ALDH1A1*)	P48644	Po. anti-bovine Abcam ab23375	1/500
Triosephosphate isomerase (*TPI1*)	Q5E956	Po. anti-human Novus NBP1-31470	1/50,000
Phosphoglycerate kinase 1 (*PGK1*)	Q3T0P6	Po. anti-human Abcam ab90787	1/5000
Fructose-bisphosphate aldolase (*ALDOA*)	A6QLL8	Po. anti-human Sigma AV48130	1/4000
Glycogen phosphorylase (*PYGB*)	Q3B7M9	Po. anti-human Santa Cruz SC-46347	1/250
**Heat shock proteins**			
Alpha-crystallin B chain (*CRYAB*)	P02510	Mo. anti-bovine Assay Designs SPA-222	1/1000
Heat shock protein beta-6, Hsp20 (*HSPB6*)	Q148F8	Mo. anti-human Santa Cruz HSP20-11:SC51955	1/500
Heat shock protein beta-1, Hsp27 (*HSPB1*)	Q3T149	Mo. anti-human Santa Cruz HSP27 (F-4):SC13132	1/3000
DnaJ homolog subfamily A member 1, Hsp40 (*DNAJA1*)	Q5E954	Mo. anti-human Santa Cruz HSP40-4 (SPM251):SC-56400	1/250
Heat shock 70 kDa protein 1A, Hsp70-1A (*HSPA1A*)	Q27975	Mo. anti-human RD Systems MAB1663	1/1000
**Oxidative stress proteins**			
Peroxiredoxin-6 (*PRDX6*)	O77834	Mo. anti-human Abnova PRDX6 (M01), clone 3A10-2A11	1/500
**Structural proteins**			
Myosin light chain 1/3 (*MYL1*)	A0JNJ5	Po. anti-human Abnova MYL1 (A01)	1/1000
Myosin heavy chain-IIx (*MYH1*)	Q9BE40	Mo anti-bovine Biocytex 8F4	1/500
Troponin T, slow skeletal muscle (*TNNT1*)	Q8MKH6	Po. anti-human Sigma SAB2102501	1/4000
Titin (*TTN*)	Q8WZ42	Mo. anti-human Novocastra NCL-TITIN	1/100
Tubulin alpha-4A chain (*TUBA4A*)	P81948	Mo anti-human Sigma T6074	1/1000
**Cell death and protein binding**			
Tripartite motif protein 72 (*TRIM72*)	E1BE77	Po. anti-human Sigma SAB2102571	1/2000
Four and a half LIM domains 1 (*FHL1*)	F1MR86	Po. anti-human Sigma AV34378	1/5000

^1^ The suppliers and conditions for each primary antibody after western blotting validation are given as in Picard et al. [36].

**Table 2:** Pearson correlation analyses between the 20 protein biomarkers with WBSF values and Intramuscular fat (IMF).

Protein Biomarkers ^1^	WBSF	IMF
**Energy metabolic enzymes**		
MDH1	−0.29 ***	−0.18 *
ENO3	−0.21 **	-
ALDH1A1	+0.34 ***	+0.38 ***
TPI1	-	-
PGK1	−0.15 *	-
ALDOA	-	-
PYGB	-	-
**Heat shock proteins**		
CRYAB	+0.32 ***	+0.37 ***
HSP20 (HSPB6)	+0.21 **	+0.31 ***
HSP27 (HSPB1)	+0.28 ***	+0.22 **
HSP40 (DNAJA1)	-	-
HSP70-1A	-	-
**Oxidative stress proteins**		
PRDX6	-	+0.20 *
**Structural proteins**		
MYL1	−0.18 *	-
MYH1	−0.26 ***	−0.26 **
TNNT1	+0.22 **	+0.15 *
TTN	-	-
TUBA4A	-	-
**Cell death and protein binding**		
TRIM72	-	−0.33 ***
FHL1	+0.18 *	+0.22 **

^1^ The correlation coefficients (q = 8) in bold font highlight the protein biomarkers common to WBSF and IMF. WBSF: Warner–Bratzler shear force; IMF: Intramuscular fat. Significance: * *p* < 0.05; ** *p* < 0.01; *** *p* < 0.001.
